# Impact of Work-Related Musculoskeletal Diseases on Quality of Life among Tailors in Tunisia

**DOI:** 10.1192/j.eurpsy.2026.11533

**Published:** 2026-07-31

**Authors:** A. Chouchane, R. Moncer, F. Barkallah, A. Aloui, A. Ghnim, I. Kacem, S. Ben Fredj, M. Makhloufi, M. Bouhoula, A. Brahem, H. Kalboussi, O. ElMaalel, S. Chatti

**Affiliations:** 1 Occupational Medicine and Professional Pathologies Department, Farhat Hached Teaching Hospital; 2 Faculty of Medicine of Sousse, Research Laboratory LR19SP03, University of Sousse; 3 Department of physical medicine and rehabilitation, Sahloul Teaching Hospital; 4 Department of Epidemilogy, Farhat Hached Teaching Hospital, Sousse, Tunisia

## Abstract

**Introduction:**

Work-related musculoskeletal disorders (WMSDs) are a major occupational health problem, often leading to pain, disability, and reduced productivity. Tailors are particularly vulnerable due to prolonged sitting and repetitive tasks, which may significantly affect their quality of life.

**Objectives:**

To evaluate the effect of WMSDs on tailors’ quality of life in a clothing factory in Tunisia.

**Methods:**

Cross sectional study conducted in collaboration with the department of occupational medicine and the department of physical medicine and rehabilitation in teaching Hospitals. The study was carried out during 6 months among tailors working in a private clothing factory. Data collection included socio-demographic information, lifestyle habits, and professional details, gathered through a structured questionnaire. We used Standardized Nordic Questionnaire for Musculoskeletal Symptoms for WMSD evaluation. To evaluate symptoms and quality of life of participants we used Musculoskeletal Health Questionnaire (MSK-HQ) .

**Results:**

A total of 130 tailors were included in the study. The mean age was 43.9±12.3 years. All participants were women. The prevalence of WRMSDs in this population was 87%. The neck involvement was the most common (69.2%). The mean score of MSK-HQ was 44.2±9.7. Eighty women (61.5%) had poor quality of life (MSK-HQ score < 50). Results showed that an increase in the number of joints affected was associated with a significant decrease in the MSK-HQ score.

**Conclusions:**

This study highlights the high prevalence of WMSDs among Tunisian tailors and their substantial negative impact on quality of life. These findings underscore the need for preventive occupational health measures and ergonomic interventions in clothing factories.

**Disclosure of Interest:**

None Declared

